# Supplementary figures and images for: The association between frailty, care receipt and unmet need for care with the risk of hospital admissions
Source: PLoS One. 2024 Sep 27;19(9):e0306858. doi: 10.1371/journal.pone.0306858 (PMC11432830; doi:10.1371/journal.pone.0306858)

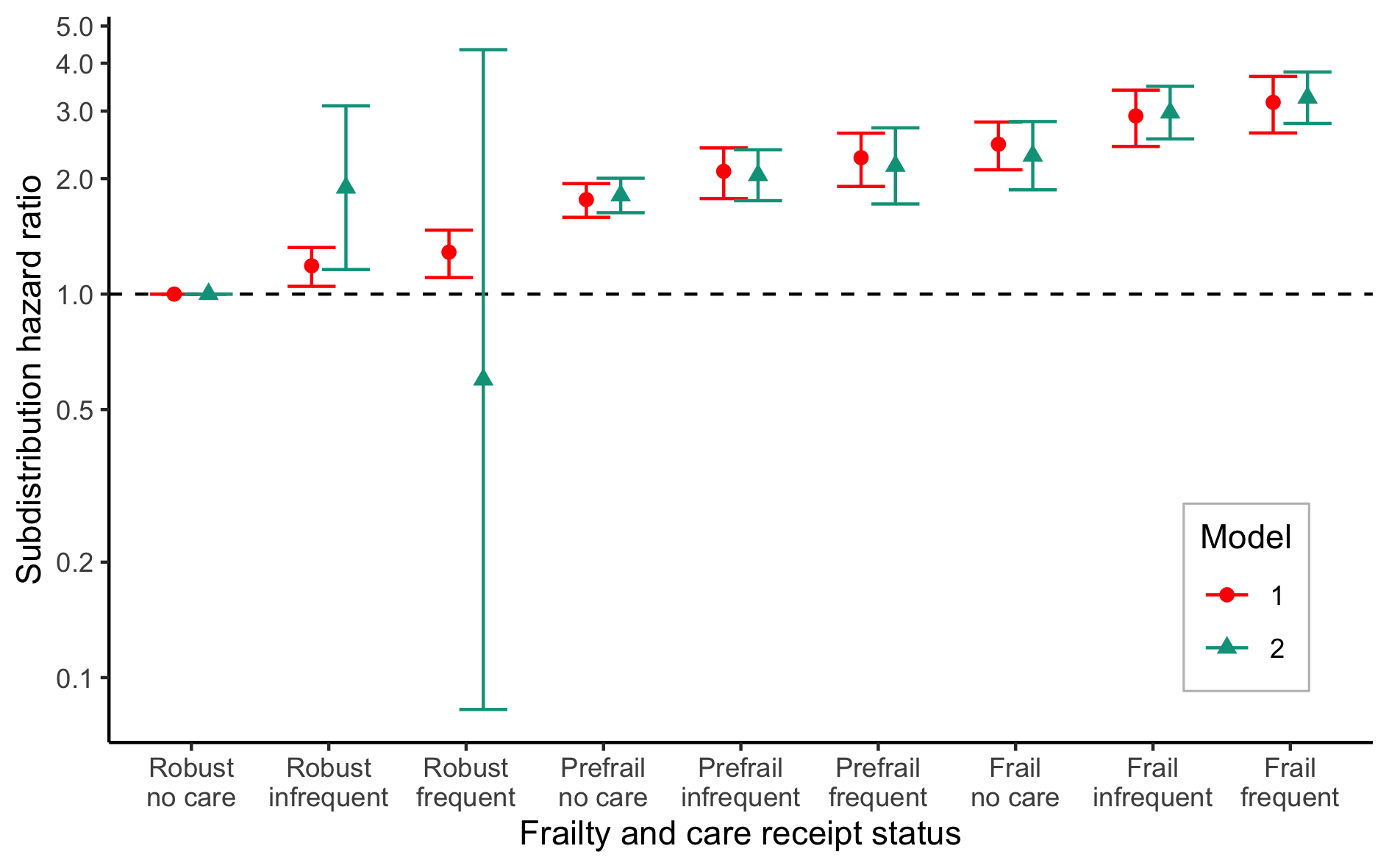

Supplement: S1 Fig — (TIF) [file pone.0306858.s001.tif]

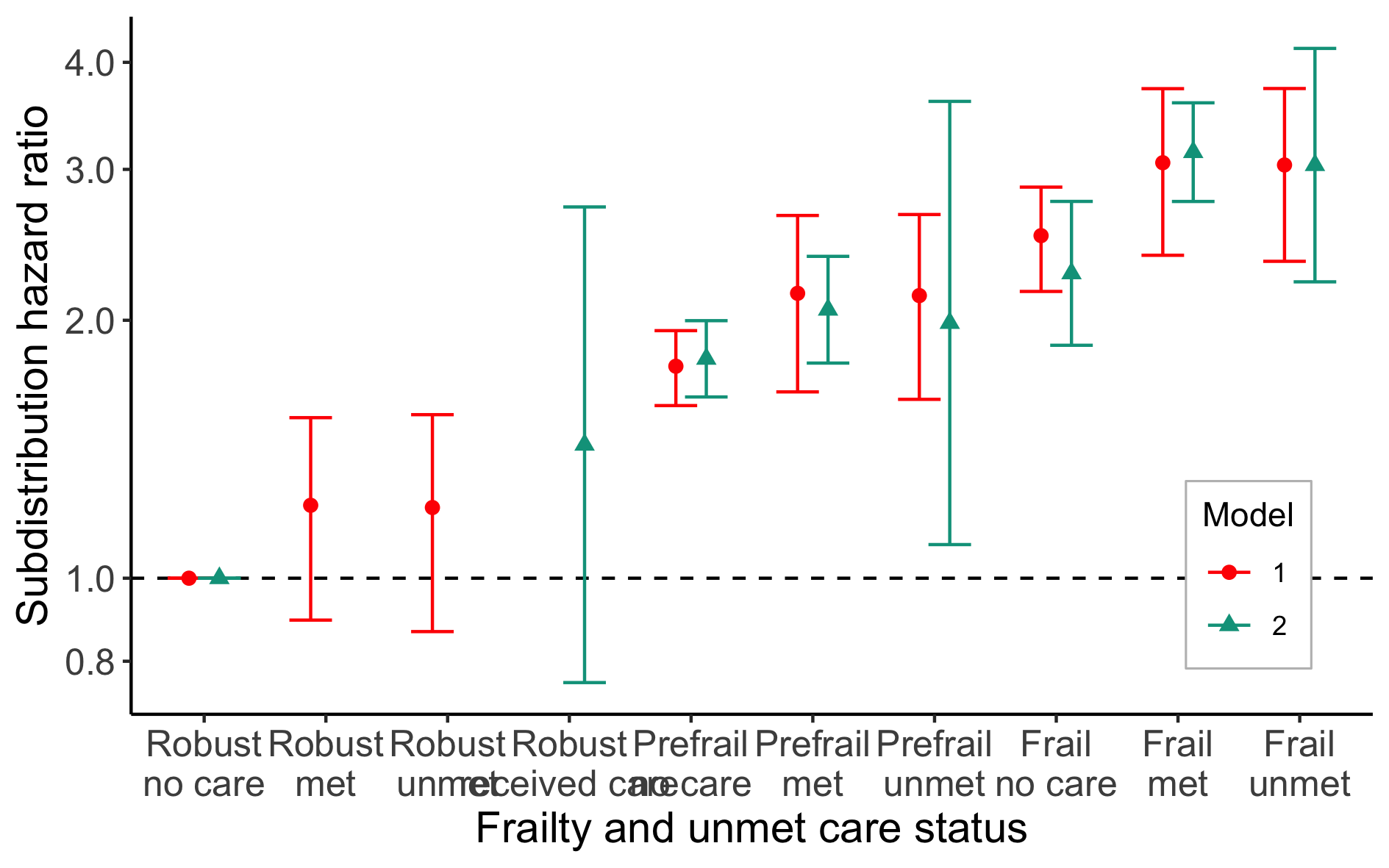

Supplement: S2 Fig — (TIF) [file pone.0306858.s002.tif]

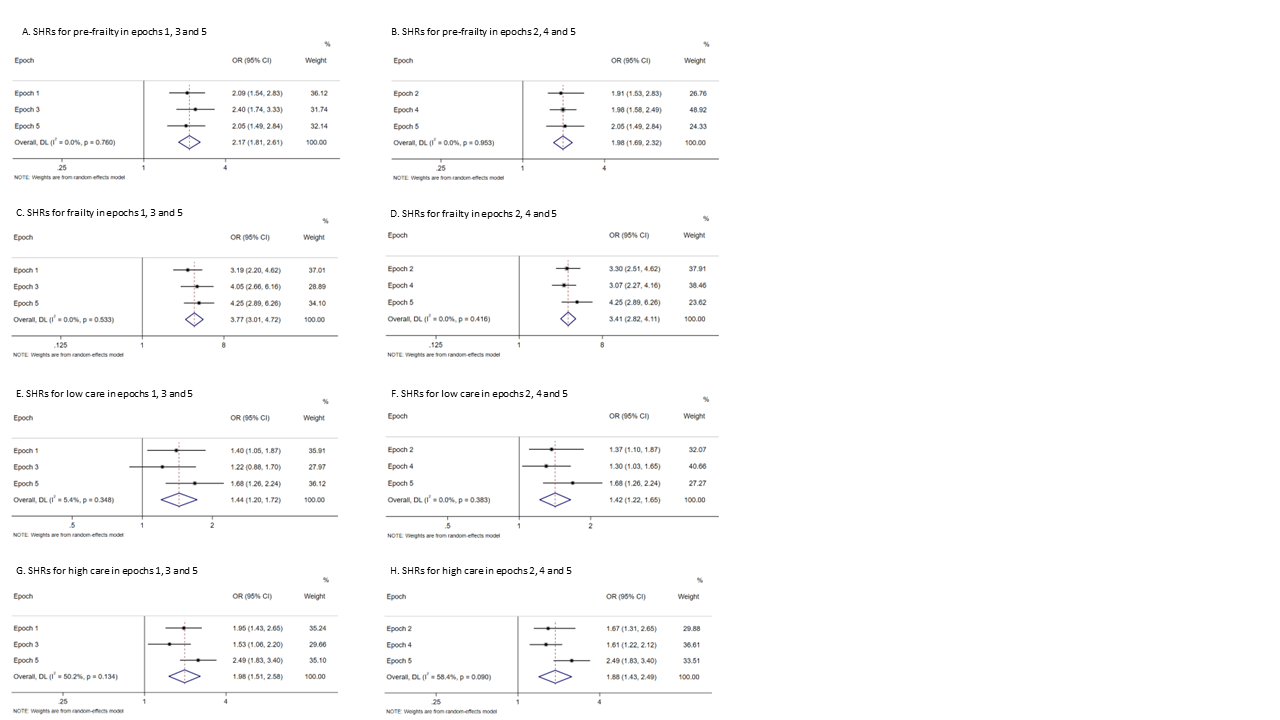

Supplement: S3 Fig — (TIF) [file pone.0306858.s003.tif]

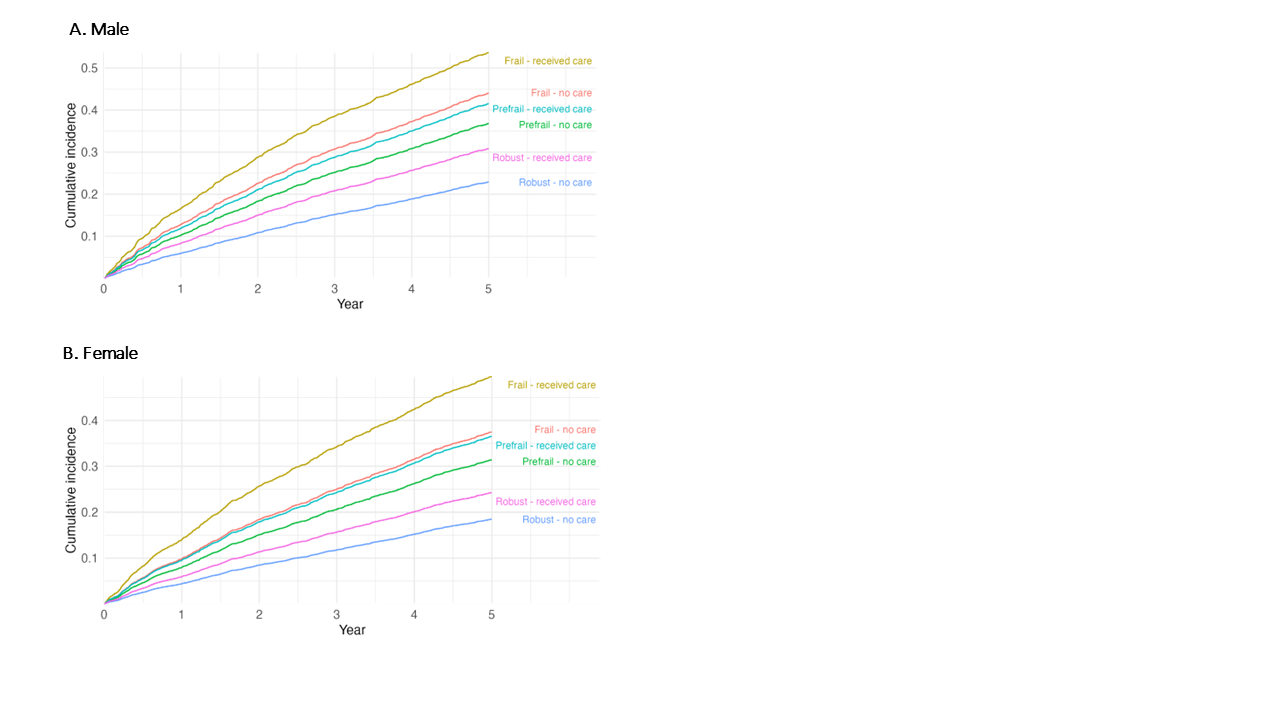

Supplement: S4 Fig — Death was the competing risk. (TIF) [file pone.0306858.s004.tif]
