# Supplementary material for: The association between frailty, care receipt and unmet need for care with the risk of hospital admissions
Source: PLoS One. 2024 Sep 27;19(9):e0306858. doi: 10.1371/journal.pone.0306858 (PMC11432830; doi:10.1371/journal.pone.0306858)
Supplement: S1 Table — (DOCX) [file pone.0306858.s005.docx]

**S1 Table. Deficit variables included in the ELSA Frailty Index**

| **Description** | **Assigned values (1 indicates a deficit, 0 no deficit)** | | | | | |
| --- | --- | --- | --- | --- | --- | --- |
| 1. Difficulty with walking 100 yards | No=0 | Yes=1 |  |  |  |  |
| 1. Difficulty sitting for about two hours | No=0 | Yes=1 |  |  |  |  |
| 1. Difficulty getting up from a chair after sitting for long periods | No=0 | Yes=1 |  |  |  |  |
| 1. Difficulty climbing several flights of stairs without resting | No=0 | Yes=1 |  |  |  |  |
| 1. Difficulty climbing one flight of stairs without resting | No=0 | Yes=1 |  |  |  |  |
| 1. Difficulty stooping, kneeling, or crouching | No=0 | Yes=1 |  |  |  |  |
| 1. Difficulty reaching or extending arms above shoulder level | No=0 | Yes=1 |  |  |  |  |
| 1. Difficulty pulling or pushing large objects like a living room chair | No=0 | Yes=1 |  |  |  |  |
| 1. Difficulty lifting or carrying weights over 10 pounds, like a heavy bag | No=0 | Yes=1 |  |  |  |  |
| 1. Difficulty picking up a 5p coin from a table | No=0 | Yes=1 |  |  |  |  |
| 1. Difficulty dressing, including putting on shoes and socks | No=0 | Yes=1 |  |  |  |  |
| 1. Difficulty walking across a room | No=0 | Yes=1 |  |  |  |  |
| 1. Difficulty bathing or showering | No=0 | Yes=1 |  |  |  |  |
| 1. Difficulty eating, such as cutting up your food | No=0 | Yes=1 |  |  |  |  |
| 1. Difficulty getting in or out of bed | No=0 | Yes=1 |  |  |  |  |
| 1. Difficulty using the toilet, including getting up or down | No=0 | Yes=1 |  |  |  |  |
| 1. Difficulty using a map to figure out how to get around in a strange place | No=0 | Yes=1 |  |  |  |  |
| 1. Difficulty preparing a hot meal | No=0 | Yes=1 |  |  |  |  |
| 1. Difficulty shopping for groceries | No=0 | Yes=1 |  |  |  |  |
| 1. Difficulty making telephone calls | No=0 | Yes=1 |  |  |  |  |
| 1. Difficulty taking medications | No=0 | Yes=1 |  |  |  |  |
| 1. Difficulty managing money, (e.g. paying bills and keeping track of expenses) | No=0 | Yes=1 |  |  |  |  |
| 1. Difficulty doing work around the house or garden | No=0 | Yes=1 |  |  |  |  |
| 1. Self-reported general health | Excellent=0 | V.good-0.25 | Good=0.5 | Fair=0.75 | Poor=1 |  |
| 1. Whether respondent has felt depressed much of the time during the past week | No=0 | Yes=1 |  |  |  |  |
| 1. Whether respondent felt everything they did during the past week was an effort | No=0 | Yes=1 |  |  |  |  |
| 1. Whether respondent felt their sleep was restless much of the time during the past week | No=0 | Yes=1 |  |  |  |  |
| 1. Whether respondent was happy much of the time during the past week | Yes=1 | No=0 |  |  |  |  |
| 1. Whether respondent felt lonely much of the time during the past week | No=0 | Yes=1 |  |  |  |  |
| 1. Whether the respondent enjoyed life much of the time during the past week | Yes=1 | No=0 |  |  |  |  |
| 1. Whether respondent felt sad much of the time during the past week | No=0 | Yes=1 |  |  |  |  |
| 1. Whether respondent could not get going much of the time during the past week | No=0 | Yes=1 |  |  |  |  |
| 1. High blood pressure or hypertension (self-reported) | No=0 | Yes=1 |  |  |  |  |
| 1. Angina (self-reported) | No=0 | Yes=1 |  |  |  |  |
| 1. Heart attack (including MI or coronary thrombosis) (self-reported) | No=0 | Yes=1 |  |  |  |  |
| 1. Congestive heart failure (self-reported) 2. An abnormal heart rhythm (self-reported) | No=0  No=0 | Yes=1  Yes=1 |  |  |  |  |
| 1. Diabetes or high blood sugar (self-reported) | No=0 | Yes=1 |  |  |  |  |
| 1. A stroke (cerebral vascular disease) (self-reported) | No=0 | Yes=1 |  |  |  |  |
| 1. Chronic lung disease such as chronic bronchitis or emphysema (self-reported) | No=0 | Yes=1 |  |  |  |  |
| 1. Asthma (self-reported) | No=0 | Yes=1 |  |  |  |  |
| 1. Arthritis (including osteoarthritis , or rheumatism) (self-reported) | No=0 | Yes=1 |  |  |  |  |
| 1. Osteoporosis, sometimes called thin or brittle bones (self-reported) | No=0 | Yes=1 |  |  |  |  |
| 1. Cancer or a malignant tumor (excluding minor skin cancers) (self-reported) | No=0 | Yes=1 |  |  |  |  |
| 1. Parkinson's disease (self-reported) | No=0 | Yes=1 |  |  |  |  |
| 1. Any emotional, nervous or psychiatric problems (self-reported) | No=0 | Yes=1 |  |  |  |  |
| 1. Alzheimer's disease (self-reported) | No=0 | Yes=1 |  |  |  |  |
| 1. Dementia, organic brain syndrome, senility or any other serious memory impairment (self-reported) | No=0 | Yes=1 |  |  |  |  |
| 1. Self-reported eyesight (while using lenses, if appropriate) | Excellent=0 | V.good=0.2 | Good=0.4 | Fair=0.6 | Poor=0.8 | Blind=1 |
| 1. Self-reported hearing (while using hearing aid if appropriate) | Excellent=0 | V.good=0.25 | Good=0.5 | Fair=0.75 | Poor=1 |  |
| 1. Whether respondent has fallen down at all /last year /last 2years | No=0 | Yes=1 |  |  |  |  |
| 1. Whether respondent has fractured hip ever /in last 2 years | No=0 | Yes=1 |  |  |  |  |
| 1. Whether respondent has had joint replacement ever | No=0 | Yes=1 |  |  |  |  |
| 1. Identify today’s date: day of month | Yes=0 | No=1 |  |  |  |  |
| 1. Identify today’s date: month | Yes=0 | No=1 |  |  |  |  |
| 1. Identify today’s date: year | Yes=0 | No=1 |  |  |  |  |
| 1. Identify the day of the week? | Yes=0 | No=1 |  |  |  |  |
| 1. Immediate word recall (sample organized into quartiles) | 1st quintile=0 | 2nd=0.3 | 3rd=0.6 | 4th quintile=1 |  |  |
| 1. Delayed word recall (sample organized into quintiles) | 1st quintile=0 | 2nd=0.25 | 3rd=0.5 | 4th=0.75 | 5th quintile =1 |  |
| 1. Have pain while performing the walking test | Yes=0 | No=1 |  |  |  |  |
