# Supplementary material for: The association between frailty, care receipt and unmet need for care with the risk of hospital admissions
Source: PLoS One. 2024 Sep 27;19(9):e0306858. doi: 10.1371/journal.pone.0306858 (PMC11432830; doi:10.1371/journal.pone.0306858)
Supplement: S2 Table — (DOCX) [file pone.0306858.s006.docx]

**S2 Table. Hospital Episode Statistics – Method of admission categories**

| **Code** | **Method of admission** |
| --- | --- |
| 11 | Waiting list |
| 12 | Booked |
| 13 | Planned |
| 21 | Accident and Emergency |
| 22 | GP-after request of immediate admission |
| 23 | Bed bureau |
| 24 | Consultant clinic |
| 25 | Mental Health Crisis Resolution team |
| 28 | Other: A&E |
| 31 | Admitted ante partum |
| 32 | Admitted post-partum |
| 81 | Transfer of any admitted patient from other hospital provider other than in an emergency |
| 82 | Baby birth |
| 83 | Baby born outside |
| 99 | Not known |
