# Supplementary material for: The association between frailty, care receipt and unmet need for care with the risk of hospital admissions
Source: PLoS One. 2024 Sep 27;19(9):e0306858. doi: 10.1371/journal.pone.0306858 (PMC11432830; doi:10.1371/journal.pone.0306858)
Supplement: S3 Table — (DOCX) [file pone.0306858.s007.docx]

**S3 Table. ICD-10 codes for fractures**

| **Code** | **Method of admission** |
| --- | --- |
| M 484 | Fatigue fracture of vertebra |
| M 495 | Collapsed vertebra in diseases classified elsewhere |
| M 80 | Osteoporosis with pathological fracture |
| M 843 | Stress fracture, not elsewhere classified |
| M 844 | Pathological fracture, not elsewhere classified |
| M 907 | Fracture of bone in neoplastic disease |
| M 966 | Fracture of bone following insertion of orthopaedic implant, joint prothesis or bone plate |
| S 02 | Fracture of skull and facial bones |
| S 12 | Fracture of neck |
| S 22 | Fracture of rib(s), sternum and thoracic spine |
| S 32 | Fracture of lumbar spine and pelvis |
| S 42 | Fracture of shoulder and upper arm |
| S 52 | Fracture of forearm |
| S 62 | Fracture of wrist and hand level |
| S 72 | Fracture of femur |
| S 82 | Fracture of lower leg, including ankle |
| S 92 | Fracture of upper limb, except ankle |
| T 02 | Fractures involving multiple body regions |
| T 08 | Fracture of spine, level unspecified |
| T 10 | Fracture of upper limb, level unspecified |
| T 12 | Fracture of lower limb, level unspecified |
| T 142 | Fracture of unspecified body region |
