# Supplementary material for: The association between frailty, care receipt and unmet need for care with the risk of hospital admissions
Source: PLoS One. 2024 Sep 27;19(9):e0306858. doi: 10.1371/journal.pone.0306858 (PMC11432830; doi:10.1371/journal.pone.0306858)
Supplement: S4 Table — (DOCX) [file pone.0306858.s008.docx]

**S4 Table:** Descriptive characteristics of the respondents (n=6,984) by level of care in ELSA wave 6 (2012/2013).

|  | **No care** | **Receiving low levels of care** | **Receiving high levels of care** |
| --- | --- | --- | --- |
| *Age group, n (%)* |  |  |  |
| 60-64 | 1,540 (86.7) | 145 (8.2) | 89 (5.0) |
| 65-69 | 1,372 (83.1) | 159 (9.6) | 118 (7.1) |
| 70-74 | 962 (79.1) | 142 (11.6) | 112 (9.1) |
| 75-79 | 707 (70.7) | 181 (18.0) | 112 (11.2) |
| 80-84 | 407 (56.5) | 190 (26.3) | 123 (17.0) |
| 85+ | 210 (33.8) | 240 (38.7) | 170 (27.4) |
| *Gender, n (%)* |  |  |  |
| Men | 2,617 (81.1) | 306 (9.5) | 301 (9.3) |
| Women | 2,577 (68.5) | 755 (20.0) | 427 (11.3) |
| *Ethnicity, n (%)* |  |  |  |
| White | 5,039 (74.6) | 1,018 (15.0) | 690 (10.2) |
| Non-White | 154 (65.4) | 43 (18.3) | 38 (16.1) |
| *Married, n (%)* |  |  |  |
| No | 1,517 (62.2) | 629 (25.8) | 292 (11.9) |
| Yes | 3,675 (80.8) | 423 (9.5) | 436 (9.5) |
| *Education attainment, n (%)* |  |  |  |
| Less than secondary school | 1,598 (63.5) | 532 (21.1) | 384 (15.2) |
| Secondary school | 995 (79.1) | 165 (13.1) | 97 (7.7) |
| College or higher | 2,602 (80.9) | 365 (11.3) | 247 (7.6) |
| *Wealth, n (%)* |  |  |  |
| 1^st^ quintile (least wealthy) | 749 (62.5) | 250 (20.8) | 199 (16.6) |
| 2^nd^ | 978 (63.7) | 325 (21.1) | 232 (15.1) |
| 3^rd^ | 1,073 (75.7) | 212 (15.0) | 131 (9.2) |
| 4^th^ | 1,173 (82.6) | 155 (10.9) | 92 (6.4) |
| 5^th^ quintile (most wealthy) | 1,147 (86.1) | 116 (8.7) | 69 (5.1) |
