# Supplementary material for: The association between frailty, care receipt and unmet need for care with the risk of hospital admissions
Source: PLoS One. 2024 Sep 27;19(9):e0306858. doi: 10.1371/journal.pone.0306858 (PMC11432830; doi:10.1371/journal.pone.0306858)
Supplement: S5 Table — (DOCX) [file pone.0306858.s009.docx]

**S5 Table:** Descriptive characteristics of the respondents (N=6,984) by need for care in ELSA wave 6 (2012/2013).

|  | **No care** | **Met care needs** | **Unmet care needs** |
| --- | --- | --- | --- |
| *Age group, n (%)* |  |  |  |
| 60-64 | 1,553 (87.5) | 195 (11.0) | 26 (1.4) |
| 65-69 | 1,397 (84.6) | 240 (14.5) | 13 (0.7) |
| 70-74 | 977 (80.3) | 224 (18.4) | 15 (1.2) |
| 75-79 | 733 (73.3) | 247 (24.7) | 19 (1.9) |
| 80-84 | 428 (59.3) | 275 (38.1) | 18 (2.4) |
| 85+ | 228 (36.7) | 360 (58.0) | 32 (5.2) |
| *Gender, n (%)* |  |  |  |
| Men | 2,646 (82.0) | 544 (16.8) | 34 (1.0) |
| Women | 2,666 (70.9) | 1,005 (26.7) | 89 (2.3) |
| *Ethnicity, n (%)* |  |  |  |
| White | 5,153 (76.3) | 1,480 (21.9) | 115 (1.7) |
| Non-White | 159 (67.3) | 68 (29.0) | 9 (3.6) |
| *Married, n (%)* |  |  |  |
| No | 1,590 (81.8) | 767 (31.4) | 82 (3.3) |
| Yes | 3,720 (65.1) | 782 (17.2) | 42 (0.9) |
| *Education attainment, n (%)* |  |  |  |
| Less than secondary school | 1,658 (65.9) | 790 (31.4) | 65 (2.6) |
| Secondary school | 1,015 (80.7) | 223 (17.7) | 19 (1.5) |
| College or higher | 2,638 (82.0) | 536 (16.6) | 39 (1.2) |
| *Wealth, n (%)* |  |  |  |
| 1^st^ quintile (least wealthy) | 780 (65.0) | 378 (31.5) | 40 (3.3) |
| 2^nd^ | 1,011 (65.9) | 479 (31.2) | 44 (2.8) |
| 3^rd^ | 1,098 (77.5) | 297 (20.9) | 21 (1.4) |
| 4^th^ | 1,190 (83.8) | 221 (15.5) | 9 (0.6) |
| 5^th^ quintile (most wealthy) | 1,157 (86.8) | 165 (12.4) | 9 (0.7) |
