# Supplementary material for: The association between frailty, care receipt and unmet need for care with the risk of hospital admissions
Source: PLoS One. 2024 Sep 27;19(9):e0306858. doi: 10.1371/journal.pone.0306858 (PMC11432830; doi:10.1371/journal.pone.0306858)
Supplement: S6 Table — Presented are number (%). (DOCX) [file pone.0306858.s010.docx]

**S6 Table. The number of hospital admissions and death in each outcome. Presented are number (%)**

| **Analysis outcomes** | **Number (%) of participants admitted to the hospital during the follow-up** | **Number (%) of participants died during the follow-up** |
| --- | --- | --- |
| Unplanned admissions | 2663 (37.78) | 310 (4.05) |
| Admissions due to fall | 586 (7.65) | 939 (12.26) |
| Admission due to fracture | 432 (5.64) | 1020 (13.32) |
