# Supplementary material for: The association between frailty, care receipt and unmet need for care with the risk of hospital admissions
Source: PLoS One. 2024 Sep 27;19(9):e0306858. doi: 10.1371/journal.pone.0306858 (PMC11432830; doi:10.1371/journal.pone.0306858)
Supplement: S7 Table — Unplanned admissions N = 2,662, competing event deaths N = 310. (DOCX) [file pone.0306858.s011.docx]

**S7 Table. Unadjusted subdistribution hazard ratio (95% CI) for the association between frailty status, level of care, need for care and each of the covariates with unplanned admissions.**

|  | **Unadjusted SHRs (95% CIs)** |
| --- | --- |
| *Frailty status, reference: robust* |  |
| Prefrail | 1.80 (1.64; 1.97) |
| Frail | 2.74 (2.47; 3.03) |
| *Level of care, reference: no care* |  |
| Received low levels of care | 1.70 (1.55; 1.87) |
| Received high levels of care | 1.82 (1.64; 2.02) |
| *Need for care, reference: no care* |  |
| Met care needs | 1.80 (1.66; 1.95) |
| Unmet care needs | 2.07 (1.61; 2.67) |
| Age (years) | 1.06 (1.06; 1.07) |
| Women (vs Men) | 0.98 (0.92; 1.06) |
| Non White (vs White) | 1.08 (0.86; 1.36) |
| Married (vs Non married) | 0.73 (0.68; 0.79) |
| *Wealth, reference: 1^st^ quintile (least wealthy)* |  |
| 2^nd^ | 0.97 (0.87; 1.09) |
| 3^rd^ | 0.77 (0.68; 0.87) |
| 4^th^ | 0.71 (0.63; 0.80) |
| 5^th^ quintile (most wealthy) | 0.56 (0.50; 0.64) |
| Education, reference: less than high school |  |
| High school | 0.81 (0.73; 0.90) |
| College or higher | 0.73 (0.67; 0.79) |

*Note:* Unplanned admissions N=2,662, competing event deaths N=310.
