# Supplementary material for: The association between frailty, care receipt and unmet need for care with the risk of hospital admissions
Source: PLoS One. 2024 Sep 27;19(9):e0306858. doi: 10.1371/journal.pone.0306858 (PMC11432830; doi:10.1371/journal.pone.0306858)
Supplement: S8 Table — (DOCX) [file pone.0306858.s012.docx]

**S8 Table. Subdistribution hazard ratio (95% CI) for the association between frailty status, frequency for care and need for care with unplanned admissions with age group as the determinant.**

|  | **Level of care** | **Need for care** |
| --- | --- | --- |
| *Frailty status, reference: robust* |  |  |
| Prefrail | 1.75 (1.58; 1.93) |  |
| Frail | 2.48 (2.15; 2.85) |  |
| Level of care, reference: no care |  |  |
| Received low levels of care | 1.20 (1.07; 1.35) |  |
| Received high levels of care | 1.34 (1.17; 1.55) |  |
| *Need for care, reference: no care* |  |  |
| Met care needs |  | 1.25 (1.13; 1.39) |
| Unmet care needs |  | 1.26 (0.95; 1.66) |
|  |  |  |
| Age group, reference: 60-64 |  |  |
| 65-69 | 1.16 (1.01; 1.33) | 1.17 (1.02; 1.34) |
| 70-74 | 1.75 (1.53; 2.01) | 1.75 (1.53; 2.01) |
| 75-79 | 2.23 (1.95; 2.55) | 2.22 (1.94; 2.55) |
| 80-84 | 2.58 (2.22; 3.00) | 2.58 (2.22; 3.00) |
| 85+ | 3.06 (2.60; 3.60) | 3.05 (2.59; 3.59) |
| Women (vs Men) | 0.76 (0.70; 0.83) | 0.76 (0.70; 0.83) |
| Non White (vs White) | 1.25 (0.96; 1.62) | 1.26 (0.97; 1.63) |
| Married (vs Non Married) | 0.90 (0.83; 0.99) | 0.90 (0.83; 0.99) |
| *Wealth, reference: 1^st^ quintile (least wealthy)* |  |  |
| 2^nd^ | 0.90 (0.79; 1.01) | 0.90 (0.80; 1.01) |
| 3^rd^ | 0.79 (0.69; 0.89) | 0.78 (0.69; 0.89) |
| 4^th^ | 0.77 (0.67; 0.88) | 0.77 (0.67; 0.87) |
| 5^th^ quintile (most wealthy) | 0.67 (0.59; 0.78) | 0.68 (0.59; 0.78) |
| Education, reference: less than high school |  |  |
| High school | 1.04 (0.92; 1.16) | 1.04 (0.92; 1.16) |
| College or higher | 0.98 (0.90; 1.08) | 0.98 (0.90; 1.08) |
