# Supplementary material for: The association between frailty, care receipt and unmet need for care with the risk of hospital admissions
Source: PLoS One. 2024 Sep 27;19(9):e0306858. doi: 10.1371/journal.pone.0306858 (PMC11432830; doi:10.1371/journal.pone.0306858)
Supplement: S9 Table — Adjusted for age group, gender, ethnicity, marital status, wealth and education. (DOCX) [file pone.0306858.s013.docx]

**S9 Table. Subdistribution hazard ratio (95% CI) for the association between frailty status, level of care, and need for care with unplanned admissions with varying time analysis.** Adjusted for age group, gender, ethnicity, marital status, wealth and education.

|  | **Level of care** | **Need for care** |
| --- | --- | --- |
| *Frailty status, reference: robust* |  |  |
| Prefrail | 1.95 (1.73; 2.20) |  |
| Frail | 3.19 (2.72; 3.74) |  |
| Level of care, reference: no care |  |  |
| Received low levels of care | 1.19 (1.04; 1.35) |  |
| Received high levels of care | 1.41 (1.21; 1.64) |  |
| *Need for care, reference: no care* |  |  |
| Met care needs |  | 1.27 (1.13; 1.43) |
| Unmet care needs |  | 1.34 (1.01; 1.78) |
|  |  |  |
| Age | 1.04 (1.04; 1.05) | 1.04 (1.04; 1.05) |
| Women (vs Men) | 0.76 (0.69; 0.83) | 0.76 (0.69; 0.83) |
| Non White (vs White) | 1.33 (0.99; 1.79) | 1.34 (0.99; 1.81) |
| Married (vs Non Married) | 0.90 (0.81; 0.99) | 0.90 (0.81; 0.99) |
| *Wealth, reference: 1^st^ quintile (least wealthy)* |  |  |
| 2^nd^ | 0.85 (0.74; 0.97) | 0.85 (0.75; 0.97) |
| 3^rd^ | 0.80 (0.70; 0.92) | 0.80 (0.70; 0.92) |
| 4^th^ | 0.77 (0.66; 0.89) | 0.77 (0.66; 0.89) |
| 5^th^ quintile (most wealthy) | 0.68 (0.58; 0.80) | 0.68 (0.58; 0.80) |
| Education, reference: less than high school |  |  |
| High school | 1.04 (0.92; 1.18) | 1.04 (0.92; 1.19) |
| College or higher | 0.98 (0.89; 1.09) | 0.98 (0.89; 1.09) |
