# Supplementary material for: The association between frailty, care receipt and unmet need for care with the risk of hospital admissions
Source: PLoS One. 2024 Sep 27;19(9):e0306858. doi: 10.1371/journal.pone.0306858 (PMC11432830; doi:10.1371/journal.pone.0306858)
Supplement: S10 Table — Unplanned admissions N = 2,662, competing event deaths N = 310. Adjusted for age group, gender, ethnicity, marital status, wealth and education. (DOCX) [file pone.0306858.s014.docx]

**S10 Table. Subdistribution hazard ratio (95% CI) for the association between frailty status and receiving care with unplanned admissions by gender**

|  | **Male** | **Female** |
| --- | --- | --- |
| *Frailty status, reference: robust* |  |  |
| Prefrail | 1.73 (1.51; 1.98) | 1.79 (1.54; 2.09) |
| Frail | 2.39 (1.93; 2.94) | 2.57 (2.12; 3.11) |
| *Receiving care, reference: no* |  |  |
| Yes | 1.30 (1.09; 1.54) | 1.31 (1.14; 1.50) |

*Note:* Unplanned admissions N=2,662, competing event deaths N=310. ^a^Adjusted for age group, gender, ethnicity, marital status, wealth and education.
